# Retraction: Lung ultrasound score in establishing the timing of intubation in COVID-19 interstitial pneumonia: A preliminary retrospective observational study

**DOI:** 10.1371/journal.pone.0245032

**Published:** 2020-12-31

**Authors:** The PLOS ONE Editors

After publication of the Expression of Concern on this article [1, 2], the authors have not provided the underlying data that support the published results. As such we cannot clarify the concerns about the similarities between the articles [1, 3] summarised in the Expression of Concern [2] and so the *PLOS ONE* Editors retract this article.

XL agreed with the retraction but stands by the published findings. MZ, AQ, LT, and SX either did not respond directly or could not be reached.
